# Lack of Major Genome-Wide DNA Methylation Changes in Succinate-Treated Human Epithelial Cells

**DOI:** 10.3390/ijms23105663

**Published:** 2022-05-18

**Authors:** Wei Cui, Zhijun Huang, Gerd P. Pfeifer

**Affiliations:** Department of Epigenetics, Van Andel Institute, Grand Rapids, MI 49503, USA; wei.cui@vai.org (W.C.); zhijun.huang@vai.org (Z.H.)

**Keywords:** DNA methylation, 5-methylcytosine, TET proteins, dioxygenases, succinate

## Abstract

The tricarboxylic acid (TCA) metabolite, succinate, is a competitive inhibitor of dioxygenase enzymes that require alpha ketoglutarate as a cofactor. One family of dioxygenases are the ten-eleven translocation (TET) proteins, which oxidize 5-methylcytosine to promote DNA demethylation. Inhibition of DNA demethylation is expected to lead to DNA hypermethylation, at least at genomic regions at which TET proteins are engaged. We treated human bronchial epithelial cells with succinate for five days and confirmed its effect on TET protein function by observing diminished formation of 5-hydroxymethylcytosine, the first oxidation product of the TET enzymatic reaction. We then analyzed global DNA methylation patterns by performing whole-genome bisulfite sequencing. Unexpectedly, we did not observe differentially methylated regions (DMRs) that reached genome-wide statistical significance. We observed a few regions of clustered DNA hypomethylation, which was also not expected based on the proposed mechanisms. We discuss potential explanations for our observations and the implications of these findings for tumorigenesis.

## 1. Introduction

DNA methylation is perhaps the most studied epigenetic pathway. Methylation of gene control regions, such as enhancers and promoters, is generally incompatible with gene expression. In mammals, enzymatic methylation of cytosines produces 5-methylcytosine (5mC) primarily at CpG dinucleotide sequences. This reaction is carried out by DNA methyltransferase proteins encoded by three genes, *DNMT1*, *DNMT3A*, and *DNMT3B*, in combination with accessory factors [1]. Once established, 5-methylcytosine can be removed from DNA either by passive dilution accomplished by ongoing DNA replication in the absence of DNMT proteins or by an active DNA demethylation process that involves oxidation of the methyl group on 5mC. This stepwise oxidation is catalyzed by a family of DNA dioxygenases, the ten-eleven-translocation (TET) proteins, which produce 5-hydroxymethylcytosine (5hmC) as the first reaction product and then proceed with the formation of 5-formylcytosine and, ultimately, 5-carboxylcytosine [2,3,4]. The latter two modified bases are removed from DNA by a base-excision repair pathway that recreates unmethylated cytosine in DNA [3,5].

In human cancer, the TET-mediated 5mC oxidation process seems to be greatly impeded. The level of 5hmC was strongly diminished in a large fraction of individual cancers and in all types of human solid tumors analyzed [6]. However, TET proteins are mutated in only a select group of human malignancies, mostly in tumors of the hematopoietic system, where TET2 undergoes relatively frequent mutation [7]. The loss of 5hmC in tumors is not explained by enhanced cell proliferation alone; it is also seen in nonproliferating compartments of the tumors [6,8]. Therefore, it has been suspected that TET proteins are dysfunctional in tumors, perhaps at the level of the biochemical pathways they are involved in, and not because the TET genes or proteins are simply downregulated [9].

When performing their catalytic function, TET proteins use 5mC in DNA (or even in RNA) as their substrate and require the cofactors molecular oxygen, alpha-ketoglutarate (alpha-KG), iron (II), and ascorbate [10]. Alpha-KG is a normal component of the tricarboxylic acid (TCA) cycle. It can be produced from isocitrate and also via the amino acid glutamine. Several metabolites and chemical compounds have been described as inhibitors of TET protein catalytic function. The first one discovered was 2-hydroxyglutarate (2-HG) [11,12,13,14]. Specific mutations in isocitrate dehydrogenases (IDH1 or IDH2) create a neomorphic enzyme that produces 2-HG as part of an aberrant catalytic cycle [11]. IDH1 mutations endowing the enzyme with such properties, for example IDH1-R132H, are found in a few types of human cancer, such as gliomas, acute myeloid leukemias, and osteosarcomas [15]. Biochemical and genetic studies have shown that the IDH1 mutation leads to an accumulation of intracellular 2-HG. 2-HG acts as a competitive inhibitor of dioxygenase enzymes that use alpha-KG as a cofactor [12]. As a result, the demethylation of histones, which depends on several methylated lysine-specific dioxygenases, and demethylation of DNA, which depends on the TET dioxygenases, will be impeded. This abnormality is thought to be a tumor-driving event, because it results in major epigenetic perturbations. There are also a few other inhibitors of TET enzymes, which include the synthetic compound C35 [16] and the metabolite itaconate, a reaction product derived from decarboxylation of *cis*-aconitate that also has structural similarity to alpha-KG [17]. One of the end products of the enzymatic TET oxidation reaction is succinate, another TCA intermediate, which is produced from alpha-KG during the TET reaction on methylated DNA. The products of an enzymatic reaction are often inhibitory to the enzyme that produces them (product inhibition) [18].

Interestingly, heterozygous germline mutations in enzyme subunits of the TCA enzymes succinate dehydrogenase (SDH) and fumarate hydratase (FH) are predisposing towards specific human malignancies. For example, gastrointestinal stromal tumors (GISTs), renal cancers, pituitary adenomas, paragangliomas, and pheochromocytomas are found in SDH mutation carriers [19]. Heterozygous germline mutations in FH predispose to uterine fibroids and papillary renal cell cancers [20]. These mutations lead to defective enzymes and the accumulation of the two TCA metabolites succinate or fumarate, respectively [21]. These two metabolites may block the activity of alpha-KG-dependent enzymes including histone and DNA demethylases. Indeed, ectopic expression of SDH and FH mutations are associated with an increase in methylated histones, including methylated H3K4 or methylated H3K27 [22], and consequently, this may result in an aberrant epigenome. SDH mutation carriers present with a DNA hypermethylation phenotype in their paragangliomas and gastrointestinal stromal tumors [23,24].

However, using mouse models of various SDH subunit deficiencies, it has been difficult to observe tumor formation, either because of embryonic lethality or because of a lack of major tumor phenotypes in conditional knockouts, or for other reasons [25]. The mutations in SDH lead to increased cellular levels of succinate, and this effect can be mimicked by treating cells with cell-permeable succinate derivatives. Such treatment results in diminished levels of 5hmC and in increased levels of methylated histones [22]. However, whether succinate has a direct effect on DNA methylation patterns in cells has not been clear. Here, we have exposed human bronchial epithelial cells to increased levels of succinate. We performed a comprehensive analysis of DNA methylation using whole-genome bisulfite sequencing (WGBS). Unexpectedly, we observed only minor changes in DNA methylation, none of which were consistent with a DNA hypermethylation effect of succinate or were reminiscent of changes commonly observed in human cancers. We discuss the potential implications of these findings in the setting of human tumorigenesis.

## 2. Results

### 2.1. Succinate Treatment of Cells

We treated the nontumorigenic, CDK4- and telomerase-immortalized human bronchial epithelial cell line HBEC3-KT [26] with different concentrations of dimethyl-succinate, a cell-permeable derivative of succinate. Dimethyl-succinate is immediately converted to succinate by esterases once it becomes intracellular. Cell morphology and viability was assessed after treatment with various concentrations of succinate (Figure S1). Cell viability was approximately 90% with 5 mM succinate, 50–70% with 10 mM succinate, and then it dropped to 28–55% with 20 mM succinate. Based on this result, we used the 5 and 10 mM concentrations in this study.

### 2.2. Effect of Succinate on 5-Hydroxymethylcytosine and 5-Methylcytosine

**Figure 1:**
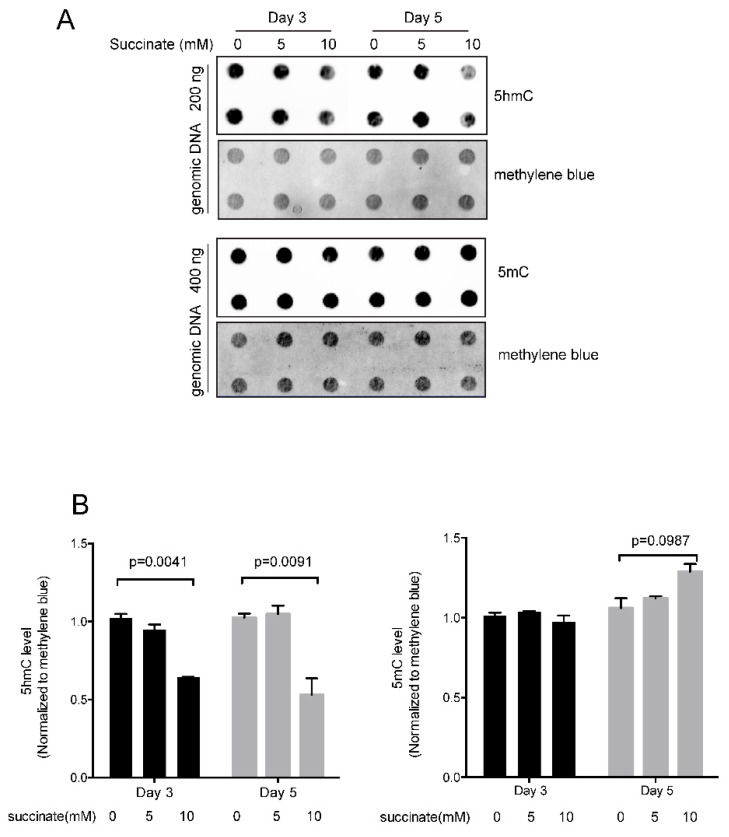
Quantitation of 5-hydroxymethylcytosine and 5-methylcytosine in succinate-treated cells. (**A**) Immuno-dot blot of 5hmC and 5mC. Bronchial epithelial cells were treated with 5 or 10 mM succinate for three days or five days. DNA was spotted onto a nylon membrane and the modified bases were detected using an anti-5hmC or anti-5mC antibody. Staining of the membrane with the DNA-binding dye methylene blue served as a loading control. (**B**) Quantitation of the 5hmC and 5mC data. A significant reduction in 5hmC levels was observed at a concentration of 10 mM succinate after three or five days. There was no significant change in 5mC levels. Three independent experiments were performed in this study.

Since succinate has been shown to inhibit dioxygenase enzymes [22], we measured the levels of 5hmC in succinate-treated cells (Figure 1). 5hmC is the first oxidation product in the reaction sequence catalyzed by TET proteins. 5hmC may persist in cells for extended periods of time in certain cellular contexts but may also be oxidized further to produce 5fC and then 5caC. These latter two bases can be removed from DNA by base-excision repair leading to a loss of DNA methylation. As expected, treatment of HBEC3-KT cells with succinate resulted in reduced levels of 5hmC. Although there was little effect at a concentration of 5 mM succinate, 10 mM succinate lowered 5hmC levels by approximately 50% (Figure 1). However, the total 5mC levels were not affected significantly after treatment with 5 or 10 mM succinate (Figure 1).

### 2.3. Comprehensive Analysis of DNA Methylation Patterns in Succinate-Treated Cells

To comprehensively analyze DNA methylation patterns genome wide, we performed whole-genome bisulfite sequencing (WGBS) of two untreated control HBEC3-KT cultures and two cell cultures treated with 10 mM succinate. We obtained between 356 and 529 million aligned reads for each sample (Table S1), reflecting a genome coverage of 22-fold on average.

**Figure 2:**
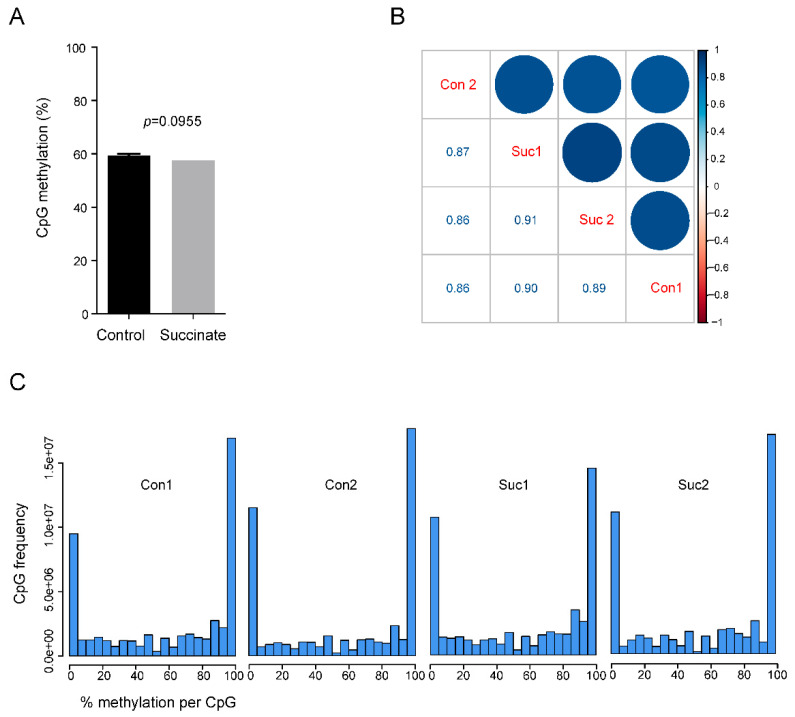
Whole-genome bisulfite sequencing of control and succinate-treated bronchial epithelial cells. (**A**) Genome-wide CpG methylation levels in control and succinate-treated bronchial cells. The bar graphs represent the % CpG methylation. (**B**) The correlogram shows the correlation of genome-wide CpG methylation between the controls (i.e., Con1 and Con2) and succinate treatment groups (i.e., Suc1 and Suc2). Sample names are shown on the diagonal. Below the diagonal are the values of Pearson correlation between samples. On the top of the diagonal, correlations are displayed in color. Color intensity and the size of the circle are proportional to the correlation coefficients. At the right side of the correlogram, the legend color shows the correlation coefficients and the corresponding colors. (**C**) The histograms show the genomic frequencies of CpG sites at each ranked methylation level (0–100% as shown on the *x*-axis) for each sample. The *y*-axis shows the frequencies of the CpG sites within the indicated range of methylation levels. Most of the sites had either high or low methylation levels.

Bisulfite sequencing does not distinguish between 5mC and 5hmC [27]. However, in cultured somatic cells, such as HBEC3-KT, the levels of 5hmC were generally one to two orders of magnitude lower than those of 5mC, and the levels of modified cytosines measured by WGBS therefore primarily reflect the 5mC base. Using the WGBS data, we first determined if there was a difference in global levels of 5mC between succinate-treated cells and controls. There was no significant difference (*p* = 0.0955); the average methylation level in control and succinate-treated cells were 59.4% and 57.5%, respectively (Figure 2A). Using pairwise comparisons, we found high degrees of similarity (R~0.9) between the control and succinate-treated samples (Figure 2B). In addition, when methylation levels, measured from 0% to 100% for each genomic CpG site, were plotted in treated versus control cells, no major differences were found (Figure 2C).

**Figure 3:**
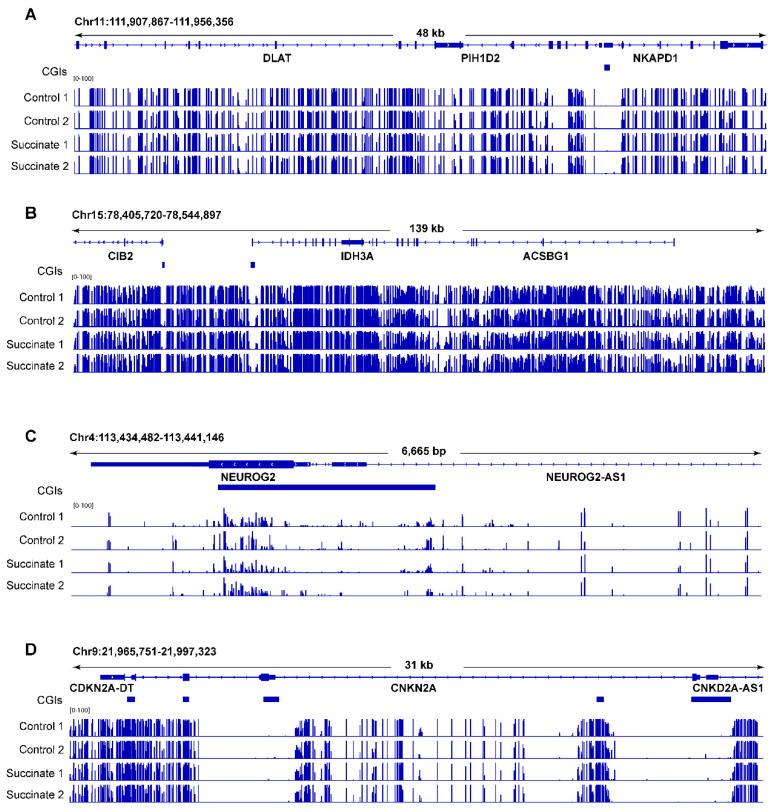
Examples of whole-genome bisulfite sequencing of control and succinate-treated bronchial epithelial cells. (**A**) A locus on chromosome 11 including the *DLAT*, *PIH1D2*, and *NKAPD1* genes; (**B**) a locus on chromosome 15 including the *CIB2*, *IDH3A*, and *ACSBG1* genes; (**C**) the *NEUROG2* locus on chromosome 4; (**D**) the *CDKN2A* locus on chromosome 9. Blue horizontal bars below the genes represent CpG islands (CGIs).

Figure 3 shows several genomic regions as examples in which no substantial DNA methylation differences can be seen. We then selected some candidate genes that are commonly methylated in human tumors, mostly homeobox genes [28], and tested them for methylation differences using combined bisulfite restriction analysis (COBRA) [29]. These regions did not display any noticeable differences in cleavage patterns (Figure S2), confirming that the methylation patterns were not changed after succinate exposure.

**Figure 4:**
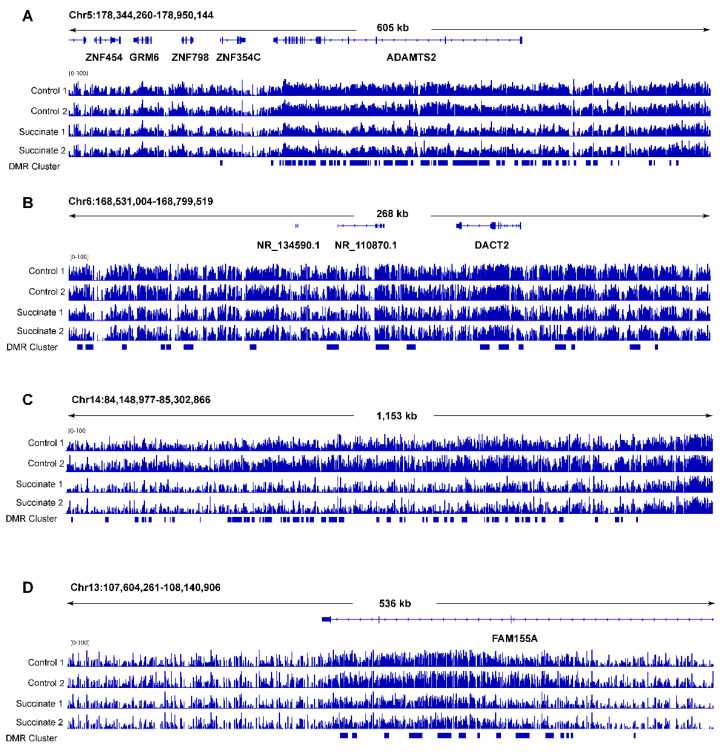
Examples of clustered DNA hypomethylated regions in succinate-treated human bronchial epithelial cells. (**A**) A locus on chromosome 5 encompassing the *ADAMTS2* gene. The clustered hypomethylated DMRs in succinate-treated cells are marked with blue bars in the bed file at the bottom of the panels. (**B**) A locus on chromosome 6 showing several hypomethylated DMRs within 250 kb as indicated by the blue bars. (**C**) A locus on chromosome 14 showing several hypomethylated DMRs as indicated by the blue bars. (**D**) A locus on chromosome 13 at the *FAM155A* gene. The clustered hypomethylated DMRs in succinate-treated cells are marked with blue bars in the bed file at the bottom of the panels.

Next, we used DMR-seq [30] to call differentially methylated regions (DMRs) in succinate-treated samples versus control samples. Contrary to expectations, this analysis did not identify any DMRs that reached genome-wide statistical significance (q < 0.05) (Table S2). When using less stringent criteria in the absence of genome-wide significance (q > 0.05 and *p* < 0.001), we were able to define a moderate number of DMRs (*n* = 2517; Table S2). These DMRs were scattered along all chromosomes in seemingly random locations. The only deviation from random occurrences were a few genomic regions (a total of approximately 10) in which hypomethylation DMRs seemed to be clustered (Figure 4). These clustered hypomethylated DMRs occurred in intragenic regions or within genes, and they were found along several chromosomes (Table S3). The occurrence of these clustered hypomethylated DMRs was not caused by lower read coverage in the succinate-treated cells at these genomic loci (Figure S3). The formation of hypomethylated DMRs in succinate-treated cells was unexpected based on the proposed model of succinate as a TET inhibitor, which should lead to DNA hypermethylation.

## 3. Discussion

Treatment of cells with succinate leads to inhibition of TET dioxygenase enzymes as reflected in a reduction in 5hmC levels. Mechanistically, this inhibition of the DNA demethylation pathway would be expected to result in increased levels of DNA methylation, as observed, for example, in paragangliomas and gastrointestinal stromal tumors [23,24]. This prediction was based on the generally well-agreed upon assumption that DNA methylation and DNA demethylation pathways are in an equilibrium and converge to a steady-state level of methylation at individual CpG sites in the genome [31,32,33]. It is assumed that inhibition of the TET family of DNA demethylase enzymes will lead to DNA hypermethylation, at least at those genomic regions in which TET proteins are actively engaged, such as promoters, enhancers, and gene bodies [4,9]. The first example of TET inhibitors promoting DNA hypermethylation was reported for the aberrant metabolite 2-hydroxyglutarate, which inhibits several dioxygenase enzymes [12]. This metabolite is formed by a mutant isocitrate dehydrogenase enzyme [14], most commonly in the form of IDH1 R132H. IDH proteins catalyze oxidative decarboxylation of isocitrate to form α-ketoglutarate, and the 2-HG metabolite competitively inhibits dioxygenases that depend on α-ketoglutarate. IDH mutations are most common in low-grade gliomas, acute myeloid leukemias, and in chondrosarcomas [34]. A subset of gliomas, those showing IDH1 mutations, are characterized by extensive DNA hypermethylation of CpG islands, a phenomenon referred to as glioma CpG island methylator phenotype (glioma CIMP) [35]. In addition, expression of this mutant IDH1 in astrocytes can recapitulate the DNA hypermethylation phenotype [36] providing evidence for a causal relationship between mutant IDH1, the 2-HG metabolite, and altered DNA methylation patterns.

Succinate, like 2-HG, is described as a competitive inhibitor of α-ketoglutarate-dependent dioxygenases [12,22]. However, the connection of succinate to altered DNA methylation patterns is not straightforward. Succinate dehydrogenases are tumor suppressors mutated in familial cancer syndromes [37]. Prior studies have shown that paragangliomas, pheochromocytomas, and gastrointestinal stromal tumors from patients with inherited mutations in *SDH* genes, particularly *SDHB*, have increased DNA methylation in their tumors [23,24]. These patterns partially resemble methylation patterns found in IDH1-mutated gliomas, although only 17 genes were concordantly hypermethylated and downregulated in both tumor types [24]. Inactivation of SDHB in mouse chromaffin cells also resulted in increased levels of succinate and in substantial hypermethylation of CpG islands [24]. To directly link the mutations and altered DNA methylation patterns mechanistically, it would seem logical to think that succinate could directly alter DNA methylation patterns in cells. However, using cell-permeable succinate, we were not able to demonstrate this connection. We were also not able to find other support in the literature showing that succinate by itself can change DNA methylation patterns. On the other hand, succinate can decrease levels of 5hmC in cultured cells or organoids [38,39] (see Figure 1). The cell-permeable succinate metabolite has been shown to increase the levels of methylated histones in succinate-exposed cells, including H3K4me1, H3K4me3, H3K27me2, and H3K79me2 [22], implying that succinate effectively inhibits histone demethylases of the dioxygenase family intracellularly. That leaves us with the question: why is DNA methylation not changing after treatment of cells with this TET inhibitor?

One possible explanation is that the treatment time of the cultured cells is not long enough. We used similar succinate concentrations and exposure times as were used in a previous study [22] in which changes in histone methylation were clearly apparent, and we also observed a reduction in the 5mC oxidation product, 5hmC, under our conditions (Figure 1). Almost all SDH-mutant human tumors carry germline mutations in SDH subunit genes. Although these are heterozygous mutations, there is a long time period before epigenome modifications may be changed at the onset of or during progression of tumorigenesis. The same extended timeframe may exist for the *Sdhb* gene-inactivated mouse cell clones [24]. Perhaps, DNA methylation is less dynamic in the bronchial cell type that we used so that even in the presence of TET inhibition, the steady-state levels of CpG methylation may be more difficult to change.

One unresolved question is why patients with germline mutations in SDH subunits are susceptible to only a handful of rare tumor types. If consistent alterations of the epigenome were at the root of tumorigenesis due to heterozygous SDH inactivation, one would expect to see a much broader spectrum of malignancies arising in these individuals. The suspected cells of origin of paragangliomas/pheochromocytomas are cells of neural crest embryonic provenance. The cells of origin of GISTs are thought to be derived from the mesenchymal lineage. These cell types are different from the epithelial cells we used in the present study. We speculate that DNA hypermethylation events in the SDH-mutant tumor types require an additional, perhaps, cell type-specific cooperating event that permits DNA hypermethylation to occur in conjunction with TET inhibition. The nature of this cooperating event is currently unknown, but it may be absent in epithelial cell types.

## 4. Materials and Methods

### 4.1. Cell Culture and Dimethyl Succinate Treatment

The HBEC3-KT cells were obtained from ATCC and were cultured in keratinocyte SFM medium (Thermo Fisher, Waltham, MA, USA, 17005042). The cells were exposed to 5, 10, or 20 mM of cell-permeable dimethyl-succinate (Sigma, St. Louis, MO, USA, W239607) for 3 or 5 days. The culture media supplemented with the same concentrations of succinate were replenished every 24 h.

### 4.2. Cell Viability Assay

The cells were seeded at a concentration of 5 × 10^4^ cells per well in 150 µL culture medium containing various concentrations of succinate. After different exposure times, 15 µL of 3-(4,5-dimethylthiazol-2-yl)-2,5-diphenyltetrazolium bromide (MTT) solution (final concentration: 0.5 mg/mL) was added to each well for 4 h; then, 150 µL of DMSO was added to each well. The absorbance was measured at a wavelength of 490 nm.

### 4.3. DNA Isolation and DNA Methylation Analysis

Genomic DNA was extracted using a Quick-DNA Miniprep Plus kit (Zymo Research, Irvine, CA, USA, D4070). The bisulfite conversion was performed with the EZ DNA Methylation-Gold Kit (Zymo Research, Irvine, CA, USA, D5005) according to the manufacturer’s instructions. COBRA assays for gene-specific DNA methylation analysis were performed according to a published method using digestion with a BstUI restriction enzyme (5’CGCG) [29]. PCR primer sequences for amplification of target regions in bisulfite-treated DNA are shown in Table S4.

### 4.4. hmC and 5mC Detection by Dot Blot Analysis

Genomic DNA isolated from control and dimethyl succinate-treated cells was incubated first with ribonuclease A, then sonicated and purified using QIAquick PCR purification kits (Qiagen, Hilden, Germany, 28104). The purified DNAs were denatured at 100 °C for 10 min and then immediately chilled on ice for 10 min and spotted onto nylon membranes. The blotted membranes were ultraviolet cross-linked, then incubated with anti-5hmC antibody (1:8000; Active Motif, 39769) or with anti-5mC antibody (1:1000; Active Motif, 39649). Following detection of the modified bases, blots were rinsed and incubated for 10 min in 0.02% methylene blue to assess total DNA levels. The changes in signal were calculated for three biological replicates by ImageJ software.

### 4.5. Whole-Genome Bisulfite Sequencing

For whole-genome bisulfite sequencing (WGBS) library preparation, genomic DNA was isolated from control cells and from 10 mM succinate-treated cells (biological duplicates each). The Accel-NGS Methyl-Seq DNA Library Kit (Swift Biosciences, Ann Arbor, MI, 30024) was used according to the manufacturer’s instructions to perform bisulfite conversion and library preparation. Sequencing was performed with an Illumina NovaSeq 6000 system with 150 bp paired-end read runs. Deduplicated reads are listed in Table S1.

### 4.6. WGBS Data Analysis

Generally, all libraries displayed high Q-scores (>30) in both reads’ pairs. We obtained data corresponding to approximately 22x genome coverage on average. Paired-end sequencing reads were aligned to the hg19 human genome using bismark [40]. Adaptors and low-quality reads were trimmed using the parameters as described previously [41].

To identify differentially methylated regions (DMRs), we used DMRseq version 0.99.0 [30]. Briefly, CpG methylation values were called by the bismark methylation extractor script provided with Bismark, using the parameters as described previously [41]. Sequencing depth for CpGs with at least three covering reads for each sample were considered for DMR calling, as well as for the percentage CpG methylation distribution and correlation analysis between samples. A single CpG coefficient cutoff of 0.05 was used for candidate regions. Significant DMRs between the control and succinate treatment were identified using q < 0.05.

The percentage CpG methylation distribution and correlation between samples were calculated by the methylKit R package [42], and a correlogram was plotted with the R package corrplot [43].

### 4.7. Statistical Analysis

Statistical analysis was performed with a two-tailed unpaired student’s *t*-test using GraphPad Prism software. The number of replicates can be found in the figure legends. Data are reported as the mean ± SEM. The *p*-values are indicated in the figures.

## 5. Conclusions

Using comprehensive whole-genome bisulfite sequencing, we showed that the TCA metabolite succinate, which is a reaction product of the 5mC oxidation reaction catalyzed by TET proteins, did not have the capacity to change DNA methylation patterns in cultured human epithelial cells as would be expected from a direct inhibitory action of this compound on TET proteins. We propose that additional or perhaps more indirect mechanisms play a role in explaining the DNA hypermethylation phenotype of succinate dehydrogenase mutant tumors.

## Data Availability

All authors had full access to all data in the study and assume responsibility for the integrity of the data and the accuracy of the data analysis. The data presented in this study are available upon request from the corresponding author. The data for genome-wide methylation analysis were deposited in the GEO database (GEO accession number: GSE200498).

## Supplementary Materials

The following supporting information can be downloaded at: https://www.mdpi.com/article/10.3390/ijms23105663/s1.
