# Supplementary material for: Lack of Major Genome-Wide DNA Methylation Changes in Succinate-Treated Human Epithelial Cells
Source: Int J Mol Sci. 2022 May 18;23(10):5663. doi: 10.3390/ijms23105663 (PMC9147453; doi:10.3390/ijms23105663)
Supplement: Supplementary file 1 [file ijms-23-05663-s001.zip › ijms-1700812-supplementary/ijms-1700812- supplementary/ijms-1700812-supplementary.pdf]

Supplementary Materials

Cui et al.

Lack of major genome-wide DNA methylation changes in succinate-treated human epithelial cells

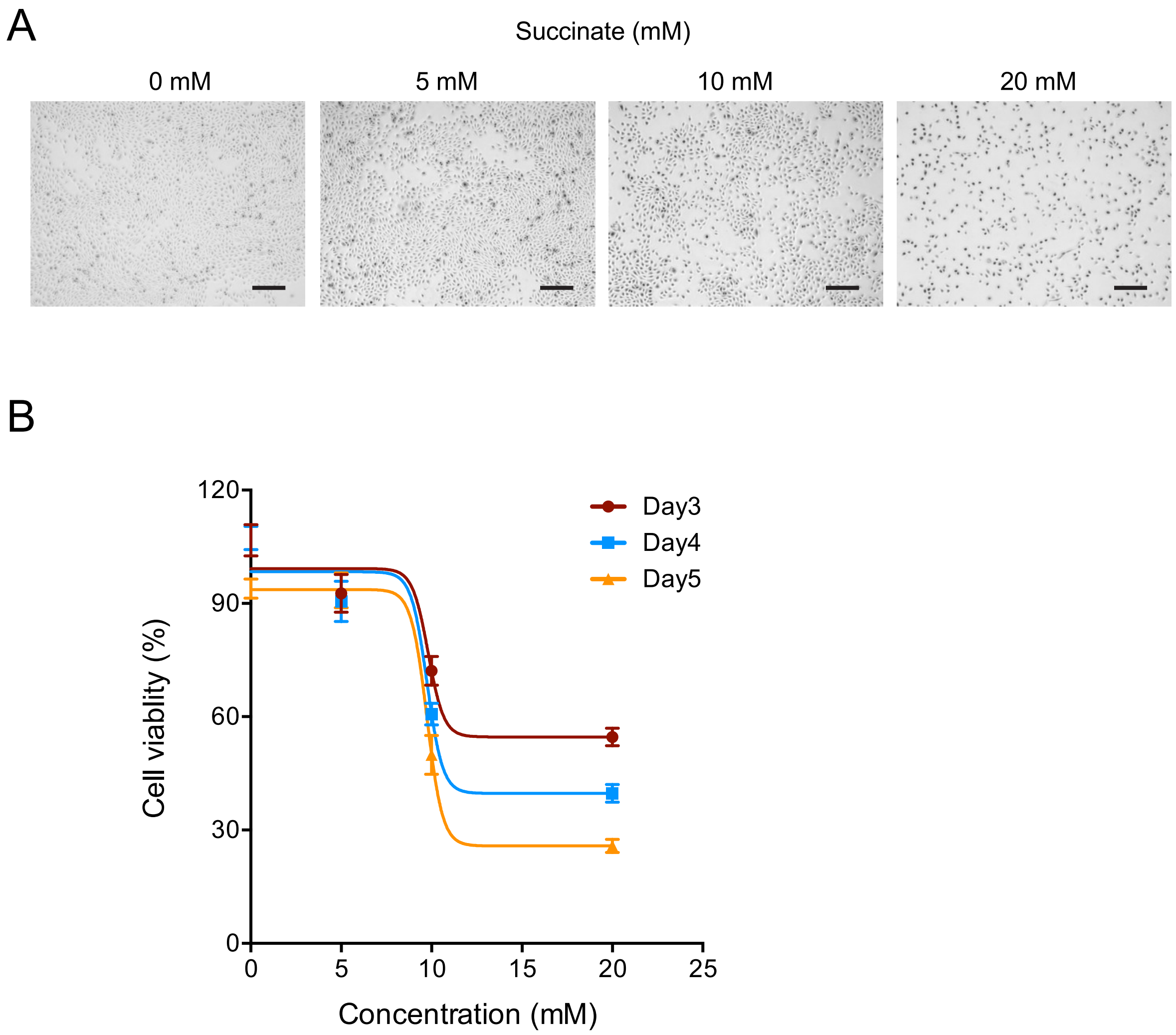

Figure S1:
Cell morphology and cell viability assay.
A. Cell morphology of human bronchial cells after 5 days of treatment with the indicated concentrations of dimethyl-succinate. Analysis was performed by light microscopy. Scale bars represent 250 μm.
B. The cells were treated with succinate at various concentrations for 3, 4 or 5 days, and cell viability was determined using the MTT assay. The results revealed dose-and time-dependent decrease in cell viability compared to untreated cells. Data are presented as the mean ± SE, n=8.

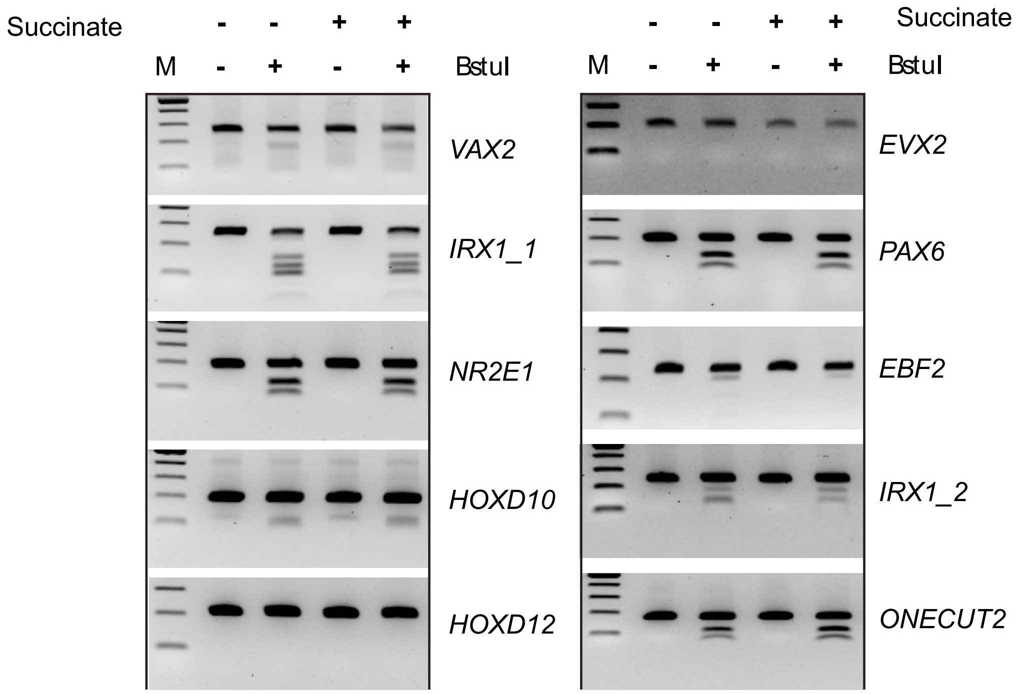

Figure S2:
DNA methylation analysis using combined bisulfite restriction analysis.
Several gene targets were analyzed by PCR amplification from bisulfite-treated DNA followed by cleavage with BstUI enzyme, which cleaves 5'CGCG sequences, but only when both cytosines are methylated and resistant to bisulfite-induced deamination. There was no difference in cleavage patterns between control and succinate-treated cells. PCR primers are listed in Table S4.

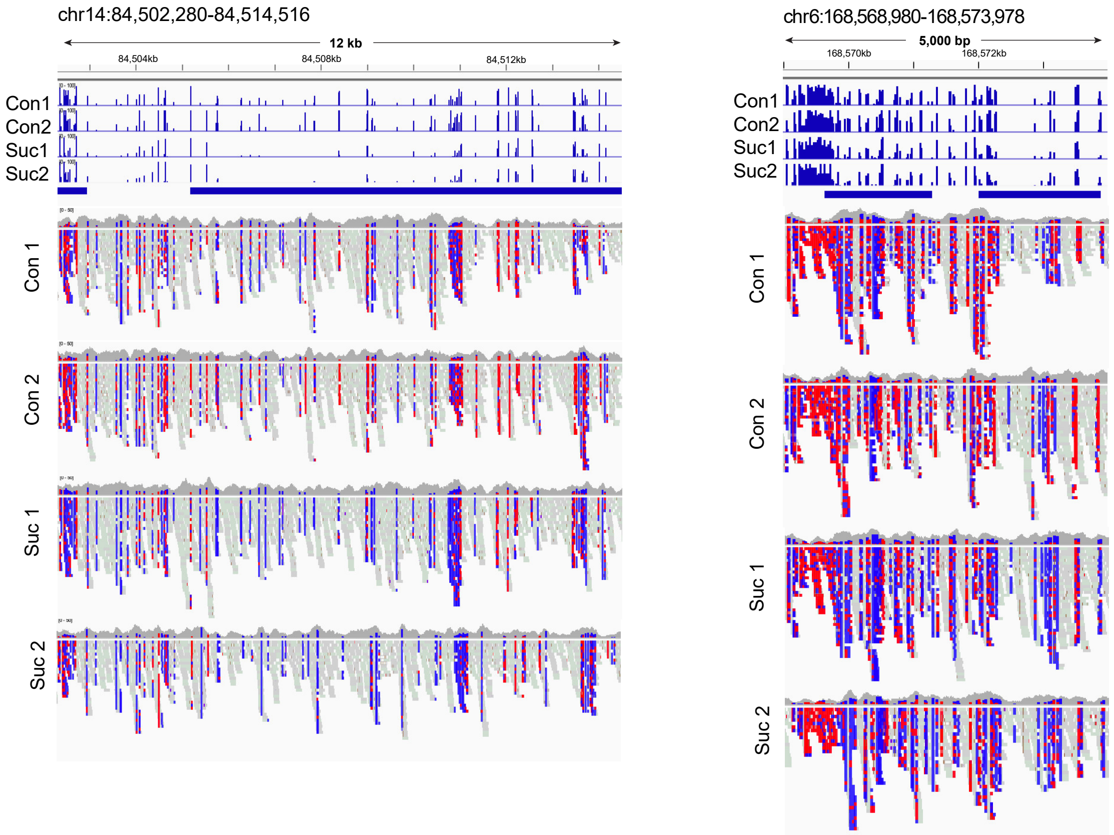

Figure S3:
Read coverage of a 5 kb and 12 kb region showing examples of hypomethylated DMRs in control and succinate-treated cells.
The aligned read tracks were taken from the Integrated Genomics Viewer (IGV) in the bisulfite mode where red represents unconverted cytosines and blue represents converted cytosines at CpG sites. The coverage has similar depth between control and succinate-treated cells. Therefore, the DMRs are not likely confounded by sequencing depth. The hypomethylated DMRs in succinate-treated cells are marked with blue bars.

Table S1: Read numbers and genome coverage.

	Control1	Control2	Succinate1	Succinate2
deduplicated read numbers	469,000,000	436,000,000	530,000,000	356,000,000
sequencing coverage	23.4	21.8	26.5	17.8

Table S2: DMRs in succinate-treated cells.

See Excel file.

Table S3: Clustered hypomethylated DMRs

chr	coordinates (kb)	Cluster size (kb)	Number of DMRs
3	88,790-89,320	530	13
4	23,794-24,313	518	23
5	178,534-178,844	310	39
6	168,454-168,771	317	20
8	108,290-108,430	140	10
10	20,301-20,541	240	10
11	23,629-23,986	357	18
13	107,830-108,025	195	13
14	84,270-85,170	900	52
22	17,718-17,912	194	12

The genomic coordinates, length of the region with hypomethylated DMRs and the number of individual DMRs in each region are indicated.

Table S4: PCR primers used for amplification of targets from bisulfite-treated DNA

Genes	Forward 5'-3'	Reverse 5'-3'
EVX2	GTTTAAGGAGTAGAATTGGAAGGTA	AAAAAAAACAATAACAAAAATAACC
PAX6	TTGAGTTTTAAGGGGAGAGTTTAGT	TAAAAAAAATCCCCAACACCTTAT
ONECUT2	AGGAGTATAGAAATTTTTTGTGTGG	ACCTAAACATTTTACCCCTCTAAAC
HOXD12	GGGGTAAAAGTGATATTGTTTAGGTTA	TACCCAAATATCCCCTAAAACTCTT
IRX1_2	GGGTTTTAATAGTTGAGGATTGTTT	CCTAACCTCCTACCTTCACAATAAC
NR2E1	TGGAGATATTATAGGGGATTTAGTT	AACATACAAAATATAAACAATCTCTACC
IRX1_1	TTTTTTTAGGTTTAGGGAGG	AAACAAACAAAAAACTTATATAAAAC
EBF2	TGGTTTGTTTAGGTTGATAGGAAGT	ATCAATAACCCCAATCCCTAAAT
HOXD10	TTTGGGGTGGGATGTTTTAG	ATCTCAACTCTAAAAACCTCCAAATAA
VAX2	GATAAATTTGTGTTTTTGTTTTTGT	CCTATCTACATTATAAATATAACCCTACC
